# Frequency of impaired fasting glucose in first degree relatives of Type-II diabetic patients and its association with Body Mass Index

**DOI:** 10.12669/pjms.36.3.57

**Published:** 2020

**Authors:** Alia Ali, Azeem Taj, Muhammed Uthman Ahmed, Elsa Tabrez

**Affiliations:** 1 Dr. Alia Ali, FCPS. Assistant Professor, Department of Medicine, Shaikh Zayed Postgraduate Medical Institute, Lahore, Pakistan; 2 Prof. Azeem Taj, Department of Medicine, Shaikh Zayed Postgraduate Medical Institute, Lahore, Pakistan; 3 Dr. Muhammed Uthman Ahmed, Associate Professor, Department of Medicine, Shaikh Zayed Postgraduate Medical Institute, Lahore, Pakistan; 4 Elsa S. Tabrez, Student, American University of Integrative Sciences, USA

**Keywords:** Impaired fasting blood sugar, BMI, Type-II diabetes

## Abstract

**Objectives:**

To determine the frequency of impaired fasting glucose in first degree relatives of people with Type-II diabetes and its association with BMI.

**Methods:**

This cross-sectional study was conducted in Diabetic clinic of Shaikh Zayed Hospital, Lahore from July to December 2017. Individuals aged ≥35 years, first degree relatives of people with Type-II diabetes, were selected and their fasting blood glucose levels were checked twice a week apart. Study participants were divided into 3 groups. Group-I were those with normal fasting blood glucose (FBS: <100mg/dl), Group-II were those with impaired fasting glucose (100-125mg/dl), considered as high risk and Group-III included those who turned out to be having frank diabetes (FBS: ≥126mg/dl). Exclusion criteria were known diabetes and pregnancy. Proportions of impaired fasting glucose levels versus BMI were compared using Chi-square test. Significance was considered at P <0.001.

**Results:**

A total of hundred subjects were included in the study with the mean age of 44.27 years. Sixty percent participants had normal FBS, 31% showed impaired FBS and 09% had frank diabetes (P <0.001). Significant association was found between impaired fasting glucose and BMI, as with increasing BMI the frequency of impaired fasting glucose increases.

**Conclusion:**

First-degree relatives of people with Type-II diabetes showed higher frequency of impaired fasting glucose and obesity was an important risk factor.

## INTRODUCTION

Diabetes mellitus is a group of metabolic diseases characterized by hyperglycemia resulting from defects in insulin secretion, insulin action or both.^1^ Type-2 diabetes mellitus is an important public health problem worldwide, and its prevalence is increasing in both developed and developing nations.^2^ Pakistan is now in the top 10 countries for absolute increase in diabetes prevalence. Prevalence of diabetes in Pakistan is high, overall prevalence is 26.3%, of which 19.2% have known diabetes and 7.1% are newly diagnosed diabetics. Prevalence of impaired fasting glucose is 14.4%.^3^ In 2019, over 19 million adults in Pakistan are estimated to be living with diabetes, putting them at risk of life threatening complications. 8.5 million of these 19 million are undiagnosed and, as a result, may be particularly at risk.^4^

Very few studies across the globe have specifically assessed the prevalence of insulin resistance in first degree relatives of Type-II diabetics, who are at increased risk for developing Type-2 diabetes in future.

It has been observed that individuals with impaired fasting glucose levels have a 20-30% chance of developing diabetes over the next 5 to 10 year.^5^ The risk factors for the development of Type-2 diabetes mellitus include family history of diabetes, increased BMI, increased waist circumference, race/ethnicity, previously identified impaired fasting glucose (IGF), or impaired glucose tolerance, history of gestational diabetes mellitus, hypertension, HDL cholesterol level <0.90mmol/L (35mg/dl) and/or triglycerides level > 2.82mmol/L (250mg/dl), polycystic ovarian syndrome and high prevalence of cigarette smoking in some south Asian populations (Bangladesh and Pakistan).^6^-^8^ There is a significant and strong association between family history of diabetes mellitus and high body mass index (BMI) with Type-2 diabetes mellitus.^9^

People with impaired glucose tolerance can reduce the risk of diabetes mellitus by changing their lifestyle, as well as by reducing their weight.^10^ Thus impaired blood glucose levels in individuals with strong family history of diabetes, particularly in first degree relatives, should be traced and their lifestyle should be modified so that the burden of diabetes mellitus and its complications may be reduced in the community. The objective of this study was to observe the prevalence of impaired fasting glucose in first-degree relatives of Type-2 diabetics and its association with BMI.

## METHODS

This is a single center observational study. This study concerns the evaluation of fasting plasma glucose in 100 subjects of Type-2 diabetic parentage coming to Diabetic Clinic, Shaikh Zayed Hospital, Lahore from July to December 2017.

### Inclusion criteria

First-degree relatives preferably siblings and children of people with Type-II diabetes were included in the study. They were all consecutive subjects 35 years or older. A cut off age of 35 at diagnosis for Type-II diabetes was commonly used to separate type 1 and Type-II diabetes in various studies.^11^ Both men and women were studied.

### Exclusion criteria

The following categories of subjects were excluded from the study:

1. Subjects with known diagnosed diabetes mellitus
2. Subjects with symptoms suggestive of diabetes mellitus such as polyuria, polydipsia, polyphagia and weight loss
3. Pregnant women because of hyperglycemia due to insulin resistance induced by placental hormones
4. Subjects suffering from liver disease
5. Subjects suffering from renal disease
6. Subjects suffering from endocrinopathies that increase blood sugar such as thyrotoxicosis, Cushing’s syndrome or disease, acromegaly
7. Subjects on drugs which may impair glucose tolerance such as glucocorticoids, thyroxin, beta adrenoceptor agonist or thiazide diuretic since these drugs may impair glucose tolerance and precipitate diabetes mellitus in susceptible subjects.

### Following information provided by all subjects was recorded in proforma

age, sex, occupation, life style (sedentary, moderate activity, hard labor or physical activity), family history of Type-2 diabetes mellitus, hypertension, and obesity.

### Weight in Kg

Weight was measured with subjects in light clothing or no clothing except underwear without shoes (secrecy being maintained in case of female subjects!).

### Height in cm

Height was measured in the standing position with the subject without shoes. It was measured from crown to the sole of feet

### BMI

BMI is derived from the formula weight/height^2^, weight being expressed in kilograms and height in meters. BMI: The normal range is 18.5-24.9 kg/m^2^. Adults are overweight if BMI is ≥ 25 kg/m^2^, pre-obese if 25-29.9 kg/m^2^ and obese if it is ≥30 kg/m^2^.

For screening, fasting is defined as no caloric intake for eight hours before the test. ADA considers an FBG level of 100mg/dl or more to be diagnostic of impaired glucose tolerance. FBG between 100-125mg/dl is called impaired fasting blood glucose and FBG 126mg/dl or more is considered as frank diabetes mellitus.

Numerical data was recorded as mean ± standard deviation (SD). Single sample X2 test was used for analysis of nominal variables. A p value of <0.05 was considered significant for all analysis. SPSS 10.0 was used for statistical analysis.

## RESULTS

Total numbers of subjects enrolled in this study were 100. We have divided the subjects into three groups on the basis of fasting blood glucose levels.

**Group-1:** (n=60), those having FBS <100mg/dl on two occasions.

**Group-2:** (n= 31), those having FBS between 100-125mg/dl on two occasions.

**Group-3:** (n=9) those having FBS ≥ 126 mg/dl on two occasions.

There were 29 males and 31 females in Group-1 with a mean age of 43.5, in Group-II there were 21 males and 10 females with a mean age of 45.9, and in Group-3 there were seven males and two females with a mean age of 43.4 in years. P-value was calculated to be 0.452, which is not significant (Table-I).

**Table-I:** Baseline characteristics of three groups.

	Group-1 (n=60)	Group-2 (n=31)	Group-3 (n=9)	P-Value
Age	Mean=43.5 SD=8.34	Mean=45.9 SD=9.70	Mean=43.4 SD=6.36	0.452
Gender	M=29 F=31	M=21 F=10	M=7 F=2	
Family H/O Obesity	n=17 (28.33%)	n=12 (38.7%)	n=7 (77.7%)	
H/O History of Smoking	n=21 (35%)	n=15 (48.3%)	n=7 (77.7%)	
Sedentary Life Style	n=21 (35%)	n=11 (35.4%)	n=3 (33.3%)	
BMI	Mean=24.76 SD+3.44	Mean=27.53 SD+4.98	Mean=29.05 SD+4.24	0.001

A positive family history of obesity was in 28.33% in Group-1, 38.70% in Group-2 and 77.77% in Group-3 respectively. A sedentary life style was present in 35% in Group-1, 35.48% in Group-2 and 33.33% in Group-3. A history of smoking was positive in 35% in Group-1, 48.38% in Group-2 and 77.77% in Group-3.

As shown in Table-I, family history of obesity is higher in Group-2 subjects as compared to Group-1. Similarly, history of smoking is again much higher in Group-2 subjects as compared to Group-1. Regarding sedentary life style, subjects in Group-2 showed slightly higher percentage as compared to Group-1. These are all significantly strong risk factors in determining the increased chances of developing diabetes mellitus in the future.

The mean BMI in Group-1 was 24.76 ±3.44, in Group-2 it was 27.53 ± 4.98 and in Group-3 it was 29.05±4.24 with a p-value of 0.001, which is considered statistically significant (Table-I).

The association of impaired fasting glucose with BMI was found to be significant as it was observed that, as the BMI increases, the chances of having impaired glucose or diabetes tend to increase.

## DISCUSSION

In our study we observed higher frequency of impaired fasting glucose in first degree relatives of people with Type-II diabetes. Out of hundred subjects with a family history of type-2 diabetes, impaired fasting blood sugar was found in 31% and frank diabetes in 9% (Table-I). A study conducted by Bock G et al., noticed that individuals with impaired fasting glucose levels have a 20-30% chance of developing diabetes over the next 5 to 10 years.^5^ Similarly, Shaikh MA et al., conducted a study which showed that individuals with positive family history of Type-2 diabetes mellitus are at high risk of developing impaired fasting blood glucose leading to a number of complications.^12^ Another study by Kumar A et al., showed that insulin resistance is observed in high prevalence in first-degree relatives of Type-2 diabetes mellitus patients; this increases as along the progression of disease is highest in first-degree relatives having Type-2 diabetes mellitus.^13^ Rodríguez-Moran M et al., also showed the presence of family history of diabetes in first degree relatives is associated with impaired fasting glucose, even in the absence of obesity.^14^

Our study also showed that history of obesity, smoking and sedentary life style has a strong impact on subjects developing impaired fasting blood sugar in future. (Table-I). Higher BMI, weight gain, dyslipidemia, hypertension and elevated fasting plasma glucose are the main predictors of progression to Type-2 diabetes mellitus.^15^ The existence of the association between Type-2 diabetes mellitus and obesity has been known for many years, and as the body fat percentage rises, the development of diabetes also rises. More so incidence of impaired FBS and diabetes was higher in those who were obese so a very clear relationship between risk of developing Diabetes mellitus in obese first degree relatives of Type-II diabetics was shown.

Our study also showed strong association between higher BMI and elevated fasting plasma glucose. (Table-II) In this study, BMI values significantly increased in the diabetic group as compared to rest of groups; this shows strong association between diabetes and elevated BMI. Park YW et al., showed associated risk factors with Type-2 diabetes that include family history of diabetes mellitus as well as increased BMI.^6^ Similarly Banerjee S et al., also showed that there is significant and strong association between family history of diabetes mellitus and high BMI.^9^

**Table-II:** Association of impaired fasting blood sugar with BMI.

	Normal FBS		Impaired FBS		Diabetes mellitus		Total	

	Count	Row N%	Count	Row N%	Count	Row N%	Count	Row N%
BMI normal	34	77.3%	9	20.5%	1	23%	44	100.0%
Overweight	23	60.5%	13	34.2%	2	53%	38	100.0%
Obese	3	16.7%	9	50.5%	6	33.3%	18	100.0%
Total	60	60.0%	31	31.0%	9	9.0%	100	100.0%

P-value of <0.001 with Chisq=24.42.

There is strong and consistent evidence that obesity management can delay the progression from prediabetes to Type-II diabetes mellitus.^16^ Life style intervention that includes dietary modifications, exercise, weight reduction and cessation of smoking, if subject is smoker, should be introduced in such patients.

Studies have shown that lifestyle intervention should be provided as a treatment option for adult prediabetic patients to improve glycemic control and reduce the prospect of their condition developing into Type-II diabetes mellitus.^17^ Increased counseling of at risk subjects by doctors/healthcare providers may also help to promote healthy behaviors to delay or prevent the onset of Type-II diabetes.^18^

### Limitation of the study

Small sample size.

## CONCLUSION

It is concluded that there is high prevalence of impaired fasting glucose in first degree relatives of people with Type-II diabetes; therefore, early screening and intervention like life style modification, of such subjects can prevent occurrence of diabetes in future as well as this will decrease national diabetic burden. Moreover, the strong association between impaired fasting glucose/diabetes and obesity suggest that one of our priorities should be maintenance of healthy weight and obesity prevention. All first-degree relatives of people with Type-2 diabetes who are overweight or obese, regardless of their blood glucose value should be counseled about lifestyle modifications.

### Authors’ Contributions:

**AA, AT, MUA:** Conceived, designed, and did statistical analysis and editing of manuscript.

**AA, MUA:** Did Data collection.

**AA, AT:** Did Review and final approval of manuscript. Dr. Alia Ali takes the responsibility for integrity of research.
